# The impact of school sports atmosphere on college students' social anxiety: the mediating role of exercise self-efficacy and the moderating effect of gender

**DOI:** 10.3389/fpubh.2026.1850192

**Published:** 2026-07-06

**Authors:** Lin Li, Jinpu Zhang, Jing Xu

**Affiliations:** 1 School of physical education, China University of mining and technology, Xuzhou, Jiangsu, China; 2 Publishing House of Chinese Journal of School Health, Xuzhou, China

**Keywords:** college students, compensatory pattern, exercise self-efficacy, gender differences, moderated mediation, school sports atmosphere, social anxiety

## Abstract

**Background:**

Social anxiety is a common psychological challenge among college students and is closely associated with impaired interpersonal functioning and reduced wellbeing. Although physical activity has been linked to better mental health, limited research has examined whether the broader school sports atmosphere is associated with social anxiety and through which psychological pathways this association may operate. Drawing on Ecological Systems Theory and Social Cognitive Theory, this study investigated whether exercise self-efficacy mediates the relationship between school sports atmosphere and social anxiety, and whether gender moderates the constituent paths of this process.

**Methods:**

A cross-sectional survey was conducted among 576 Chinese college students selected through stratified cluster sampling. Participants completed measures of school sports atmosphere, exercise self-efficacy, and social anxiety. Descriptive statistics, correlational analyses, confirmatory factor analysis, and common method bias testing were performed. Mediation and moderated mediation analyses were conducted using PROCESS Model 4 and Model 59 with bootstrapping procedures, while controlling for relevant demographic covariates.

**Results:**

School sports atmosphere was significantly and negatively associated with social anxiety. Exercise self-efficacy partially mediated this relationship, indicating that a more positive school sports atmosphere was associated with higher exercise self-efficacy, which in turn was associated with lower social anxiety. Gender significantly moderated two constituent paths of the indirect process. Specifically, the positive association between school sports atmosphere and exercise self-efficacy was stronger for males, whereas the negative association between exercise self-efficacy and social anxiety was stronger for females. However, the overall indirect effect did not significantly differ in magnitude across genders, suggesting a potential path-specific compensatory pattern rather than a universal mechanism.

**Conclusions:**

A supportive school sports atmosphere may serve as a protective contextual factor associated with lower social anxiety among college students, partly through enhanced exercise self-efficacy. The findings further suggest that the internal pathways linking sports atmosphere, self-efficacy, and social anxiety differ by gender in a compensatory manner. These results provide evidence for developing gender-sensitive campus sports and mental health interventions aimed at reducing social anxiety in higher education settings.

## 1 Introduction

College students inhabit a critical transitional developmental phase characterized by the shift from semi-structured adolescent environments to highly autonomous young adult social ecosystems. During this period, psychological wellbeing and social adaptability are paramount indicators of successful holistic development. Unfortunately, social anxiety has emerged as a pervasive negative emotional state within the college demographic. Social anxiety is clinically defined as a persistent, debilitating fear of negative evaluation, scrutiny, or judgment in social or performance situations, often leading to avoidance behaviors (1). Prolonged exposure to social anxiety not only diminishes subjective wellbeing and general life satisfaction but also substantially increases the vulnerability to co-occurring psychopathology, including clinical depression, profound loneliness, and problematic internet usage as an avoidance coping mechanism (2–4). Current epidemiological studies indicate that the prevalence of social anxiety among college students remains alarmingly high and exhibits an upward trajectory exacerbated by compounding stressors such as academic pressure, complex interpersonal dynamics, and impending occupational transitions (5–8). Consequently, systematically investigating the modifiable environmental antecedents and internal cognitive pathways that govern social anxiety is of theoretical and practical significance for establishing effective campus-based psychological interventions.

The university campus serves as the primary developmental microsystem for young adults. Within this context, the school sports atmosphere represents a critical, yet frequently under-examined, environmental variable that systematically influences students' physiological and psychological trajectories. The school sports atmosphere encompasses institutional prioritization of physical education, the comprehensiveness of the curriculum, the accessibility of sports infrastructure, the vibrancy of extracurricular sports culture, and the collective psychosocial attitudes toward physical activity among peers and faculty (9, 10). Grounded in Bronfenbrenner's (11) Ecological Systems Theory, a supportive environmental microsystem provides essential psychosocial scaffolding that promotes adaptive developmental outcomes. A robust sports atmosphere theoretically provides students with stable opportunities for physical exertion, positive emotional regulation, and structured, low-stakes social interactions, all of which may serve as protective factors against psychological distress (12). Empirical investigations have demonstrated that physical activity is negatively correlated with social anxiety and positively associated with psychological resilience (13–15). However, the direct empirical association between the broader institutional sports atmosphere and individual-level social anxiety, alongside the intricate psychological pathways bridging these constructs, remains insufficiently mapped in the current literature.

To address this empirical gap, the present study integrates Social Cognitive Theory to explore the mediating role of exercise self-efficacy. According to Bandura's (16, 17) theoretical framework, self-efficacy, the belief in one's capability to organize and execute actions required to manage prospective situations, acts as a central cognitive mediator translating environmental stimuli into behavioral and emotional responses. Exercise self-efficacy specifically denotes an individual's confidence in their ability to maintain regular physical activity despite various barriers (18, 19). An optimal school sports atmosphere provides the requisite mastery experiences, vicarious learning opportunities, and social persuasion necessary to cultivate high exercise self-efficacy. High exercise self-efficacy not only fosters adherence to physical activity but also facilitates a generalization effect, wherein increased physical confidence and emotional regulation skills transfer to social domains, thereby attenuating social anxiety (20, 21).

Furthermore, demographic heterogeneity, particularly gender, represents a critical boundary condition in understanding physical activity psychology. Research consistently highlights a gender disparity in activity levels and psychological responses to exercise environments, noting that women are generally less active than men and experience different psychosocial barriers (22–24). Socialization processes often cultivate distinct sporting identities and sensitivities to social evaluation between males and females, suggesting that the pathways connecting school sports atmosphere, exercise self-efficacy, and social anxiety may function asymmetrically across gender lines. Thus, understanding the dual roles of internal cognitive mediators and demographic moderators is essential for comprehensive theoretical advancement. Therefore, the current study constructs and tests a moderated mediation model to evaluate whether exercise self-efficacy mediates the relationship between the school sports atmosphere and social anxiety, and whether gender moderates these constituent pathways.

## 2 Theoretical framework and hypotheses development

### 2.1 School sports atmosphere and social anxiety

The ecological perspective posits that human behavior and emotional states are inextricably linked to the proximal environments they inhabit. The school sports atmosphere constitutes a multidimensional psychosocial climate that significantly dictates the quality of campus life (25). An enriched sports atmosphere functions as a protective environmental buffer. In such an environment, social interactions occur within the context of structured games, collaborative team sports, or shared physical exertion, which organically shifts the cognitive focus away from interpersonal evaluation toward collective task completion. This paradigm shift is critically important for individuals pre-disposed to social anxiety. Theoretical models of social anxiety, such as the self-presentation theory, suggest that individuals with lower self-evaluations anticipate negative scrutiny in social settings, which triggers profound anxiety (1). Engaging in a vibrant sports culture allows for low-pressure socialization, helping to interrupt the typical avoidance cycle characteristic of socially anxious individuals.

Furthermore, a supportive sports atmosphere encourages regular physical activity, which has well-documented neurobiological benefits, including the regulation of neurotransmitters that directly ameliorate physiological arousal and anxiety (15, 26, 27). Therefore, an environment that normalizes and facilitates physical activity structurally reduces the environmental precursors to social anxiety. A campus that prioritizes physical wellbeing fosters a collective ethos of resilience and stress management. Based on these theoretical deductions, we propose the following hypothesis:

*Hypothesis 1 (H1)*: the school sports atmosphere is negatively and significantly associated with college students' social anxiety.

### 2.2 The mediating role of exercise self-efficacy

While the direct environmental influence is critical, the internalization of these external factors is governed by cognitive appraisals. Bandura's (17) Social Cognitive Theory provides a robust explanatory framework for this process, emphasizing that human agency is primarily driven by self-efficacy beliefs. Self-efficacy is shaped by four primary sources: mastery experience, vicarious experience, social persuasion, and physiological states (28). A positive school sports atmosphere serves as a rich repository for these sources. Readily available facilities and inclusive curricula guarantee mastery experiences regardless of baseline athletic ability; observable peer participation provides vicarious modeling; and supportive faculty and institutional messaging serve as verbal persuasion. Consequently, a strong sports atmosphere systematically elevates an individual's exercise self-efficacy.

The subsequent transition from exercise self-efficacy to reduced social anxiety relies on the psychological principle of efficacy generalization. When students develop high confidence in their physical capabilities and their ability to overcome exercise barriers, this enhanced self-concept positively alters their broader self-esteem and resilience (29, 30). Enhanced resilience and physical self-concept directly counteract the negative self-evaluative core of social anxiety. As students experience competence in the physical domain, their anticipatory anxiety regarding social interactions diminishes. They perceive themselves as more capable of handling social complexities and are less intimidated by potential negative evaluations from peers (31, 32). Thus, the external environmental support may translate into a durable internal cognitive asset that subsequently relates to lower anxious psychopathology. We therefore propose the following hypothesis:

*Hypothesis 2 (H2)*: exercise self-efficacy significantly mediates the relationship between the school sports atmosphere and college students' social anxiety.

### 2.3 The moderating role of gender

The mediational pathways connecting environmental support, cognitive efficacy, and emotional outcomes do not operate uniformly across populations. Gender constitutes a fundamental sociocultural and biological variable that heavily influences both sports participation and the phenomenology of social anxiety. Existing literature documents significant gender disparities in both domains. Women report higher prevalence rates of social anxiety and are generally more sensitive to interpersonal evaluation and bodily appearance anxiety (24, 33). Conversely, men typically report higher levels of physical activity and greater initial exercise self-efficacy, largely due to traditional gender role socialization that aggressively promotes male participation in competitive physical endeavors (34).

These baseline disparities suggest that the strength of the constituent paths in our proposed mediation model will vary by gender. Regarding the first stage of the mediation model (from school sports atmosphere to exercise self-efficacy), males often receive more direct social reinforcement and accumulate more early mastery experiences in sports (35). Consequently, when placed in a supportive campus sports environment, males possess the foundational athletic schema to immediately leverage these resources, translating environmental opportunities into self-efficacy more efficiently than females. Therefore, we anticipate that the positive association between the sports atmosphere and exercise self-efficacy will be stronger for males.

Regarding the second stage (from exercise self-efficacy to social anxiety), the literature suggests a potential compensatory pattern. Because females constitute a higher-risk demographic for social anxiety and are more heavily impacted by negative evaluative fears regarding physical presentation, the acquisition of high exercise self-efficacy may act as a more potent psychological intervention (3). For females, overriding the barrier of physical insecurity provides a boost to general resilience, thereby yielding a steeper reduction in social anxiety compared to males, for whom physical efficacy might simply reinforce an already established baseline. Based on this synthesized rationale, we propose the following hypothesis:

*Hypothesis 3 (H3)*: gender moderates the individual constituent paths of the mediation model. Specifically, the path from sports atmosphere to exercise self-efficacy is stronger for males, whereas the path from exercise self-efficacy to social anxiety is stronger for females, suggesting a path-specific compensatory pattern.

## 3 Methods

### 3.1 Participants and procedure

**Figure 1:**
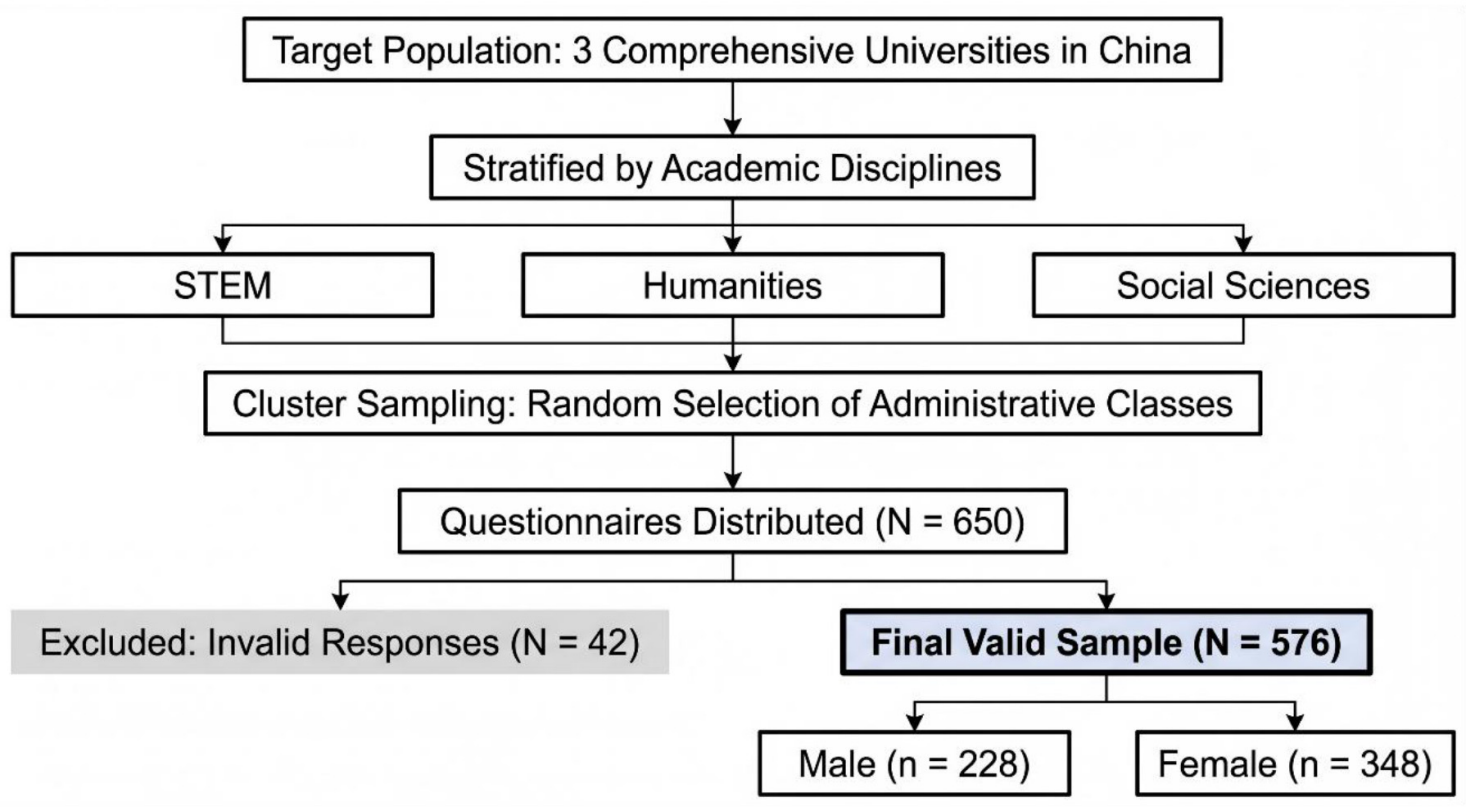
Sampling framework and participant flow diagram.

This study employed a cross-sectional quantitative design to evaluate the proposed moderated mediation framework. Data were collected between March and June 2024 utilizing a stratified cluster sampling technique. First, stratification was conducted based on academic disciplines (e.g., STEM, Humanities, and Social Sciences) to ensure sample representativeness across three comprehensive universities in China. Subsequently, within each stratum, natural administrative classes were randomly selected as clusters. All students within the selected class clusters were invited to participate. A flow diagram illustrating the sampling framework is presented in Figure 1.

Prior to survey administration, ethical approval was formally obtained from the Institutional Review Board of the host university, ensuring strict adherence to the ethical guidelines stipulated by the Declaration of Helsinki. All prospective participants were thoroughly briefed regarding the academic purpose of the research, the absolute anonymity and confidentiality of their responses, and their unconditional right to withdraw from the study at any juncture without penalty. Written informed consent was secured from all participants electronically before they were permitted to access the survey instruments.

A total of 650 questionnaires were distributed. We received 618 responses, yielding an initial return rate of 95.08%. Following a strict data screening protocol, which eliminated questionnaires exhibiting uniform response patterns, conspicuous logical contradictions, or missing data on core variable constructs, 42 invalid responses were discarded. The final sample consisted of 576 valid responses. The final valid-response rate was 88.62%. The sample comprised 228 male students (39.58%) and 348 female students (60.42%). The age of the participants ranged from 17 to 26 years, with a mean age of 20.32 years and a standard deviation of 1.57 years.

### 3.2 Measures

Participants completed a demographic questionnaire alongside three highly validated psychometric instruments designed to assess the central variables.

*(1) School Sports Atmosphere*. The perceived campus environment was evaluated using a modified version of the School Climate Measure (9), specifically adapted to evaluate physical activity contexts for Chinese college students through translation, back-translation, and expert review. This instrument comprises 18 items distributed across four core dimensions: curriculum and pedagogical atmosphere, extracurricular sports activity atmosphere, sports facility and infrastructure atmosphere, and interpersonal sports support. Responses were recorded on a 5-point Likert scale ranging from 1 (completely inconsistent) to 5 (completely consistent). Aggregate scores ranged from 18 to 90, with higher scores denoting a more positive perception of the campus sports environment. In the current investigation, the scale demonstrated excellent internal consistency, yielding a Cronbach's alpha coefficient of.91.

*(2) Exercise Self-Efficacy*. Exercise self-efficacy was measured utilizing the generalized Exercise Self-Efficacy Scale, originally conceptualized by Marcus et al. (36). This 10-item inventory quantifies an individual's subjective confidence and cognitive belief in their capacity to initiate and maintain a regimented physical exercise routine despite encountering various typical impediments, such as fatigue, lack of time, or inclement weather. Items were scored on a 5-point Likert scale ranging from 1 (not at all confident) to 5 (extremely confident). Total scores spanned from 10 to 50. This scale has been extensively validated within the college student population and exhibited robust reliability in this study, with a Cronbach's alpha of.89.

*(3) Social Anxiety*. Social anxiety was assessed employing the Interaction Anxiousness Scale (IAS) developed by Leary (37). This 15-item psychometric tool is widely regarded as a reliable standard for evaluating the subjective experience of anxiety in social interactions independent of associated behavioral avoidance. Participants rated the extent to which statements reflected their personal experiences on a 5-point Likert scale ranging from 1 (not at all characteristic of me) to 5 (extremely characteristic of me). Following the reversal of appropriate negatively worded items, total scores were calculated, ranging from 15 to 75. Higher composite scores are indicative of more severe symptomatology regarding social anxiety. The Cronbach's alpha for the IAS in the present sample was.90.

### 3.3 Statistical analysis

Data management and all statistical analyses were executed using SPSS version 26.0 and the PROCESS macro version 4.3 developed by Hayes (38). Descriptive statistics and Pearson correlational analyses were initially conducted to evaluate variable distributions and bivariate associations. Prior to hypothesis testing, structural validity and common method bias were carefully assessed using Confirmatory Factor Analysis (CFA) via AMOS 26.0.

To robustly test the mediation and moderated mediation hypotheses, ordinary least squares (OLS) regression-based path analysis was performed. Specifically, Model 4 of the PROCESS macro was utilized to determine the simple mediating effect of exercise self-efficacy, while Model 59 was deployed to evaluate the comprehensive moderated mediation model. For all regression analyses, continuous predictors defining interaction terms were mean-centered to mitigate multicollinearity. Demographic variables including age, academic grade, major, geographic origin, and exercise duration were entered as covariates to isolate the specific statistical associations of the independent variables. To calculate standard errors and ensure the robustness of the confidence intervals, non-parametric bootstrapping was employed with 5,000 resamples. Statistical significance was defined by a 95% bias-corrected confidence interval strictly excluding zero. For moderation analysis, gender was coded as a dummy variable (Male = 0, Female = 1) to establish a clear baseline for the interaction coefficients.

## 4 Results

### 4.1 Common method bias and measurement model testing

Given that all data were acquired via self-report cross-sectional methodology, the potential threat of common method bias (CMB) and the discriminant validity of the constructs were comprehensively evaluated prior to substantive hypothesis testing (39). First, Harman's single-factor test revealed the presence of multiple distinct factors possessing eigenvalues exceeding 1.0, with the first principal component explaining only 26.43% of the total variance, falling significantly below the universally accepted 40.0% threshold.

To provide a more robust assessment, a confirmatory factor analysis (CFA) was executed. The hypothesized three-factor measurement model (school sports atmosphere, exercise self-efficacy, social anxiety) demonstrated an excellent fit to the empirical data: χ^2^/df = 2.15, Comparative Fit Index (CFI) = 0.95, Tucker-Lewis Index (TLI) = 0.94, Root Mean Square Error of Approximation (RMSEA) = 0.045, and Standardized Root Mean Square Residual (SRMR) = 0.038. Importantly, this theoretical three-factor model significantly outperformed a constrained single-factor model where all items were loaded onto one latent construct (χ^2^/df = 11.42, CFI = 0.62, TLI = 0.58, RMSEA = 0.14, SRMR = 0.12; Δχ^2^ (3) = 1,452.34, *p* < 0.001). Consequently, discriminant validity is strongly supported, and common method bias does not pose a substantial threat to the validity of the findings.

### 4.2 Descriptive statistics and correlational analysis

Table 1 details the demographic profile of the participants and the corresponding descriptive statistics for the principal continuous variables, alongside the results of independent samples *t*-tests and one-way ANOVAs. The analyses revealed significant gender disparities across all main constructs. Male students reported significantly higher perceptions of the school sports atmosphere (*t* = 7.42, *p* < 0.001) and greater exercise self-efficacy (*t* = 9.15, *p* < 0.001) compared to female students. Conversely, female students exhibited significantly higher baseline levels of social anxiety (*t* = −7.12, *p* < 0.001). Additionally, age-group differences were significant across the variables, though they are not the primary focus of the present study.

**Table 1:** Demographic characteristics, variable distributions, and difference testing (N = 576).

Variables	Category	*N* (%)	School sports atmosphere	Exercise self-efficacy	Social anxiety
Gender	Male	228 (39.58)	62.35 ± 9.87	35.84 ± 7.26	35.26 ± 8.94
	Female	348 (60.42)	56.17 ± 9.62	30.26 ± 7.15	40.69 ± 8.87
	Test statistic		*t* = 7.42^***^	*t* = 9.15^***^	*t* = −7.12^***^
Age	17–18	152 (26.39)	57.24 ± 10.58	31.05 ± 8.13	40.12 ± 9.58
	19–20	284 (49.31)	58.97 ± 10.12	32.68 ± 7.64	38.76 ± 9.12
	21–22	92 (15.97)	59.36 ± 9.85	33.42 ± 7.58	37.25 ± 8.76
	≥ 23	48 (8.33)	61.25 ± 9.43	35.17 ± 7.02	35.08 ± 8.25
	Test statistic		*F* = 2.98^*^	*F* = 3.12^*^	*F* = 2.85^*^
Origin	Urban	312 (54.17)	59.03 ± 10.28	32.84 ± 7.79	38.26 ± 9.18
	Rural	264 (45.83)	58.14 ± 10.42	32.04 ± 7.86	38.87 ± 9.35
	Test statistic		*t* = 1.02	*t* = 1.22	*t* = −0.78
Total		576 (100.0)	58.62 ± 10.35	32.47 ± 7.82	38.54 ± 9.26

Data presented as mean ± SD.

^*^p < 0.05; ^***^p < 0.001.

Pearson bivariate correlational analyses were executed to investigate the unadjusted associations among the focal theoretical constructs. As detailed in Table 2, a robust positive correlation was identified between the school sports atmosphere and exercise self-efficacy (*r* = 0.526, *p* < 0.001). Furthermore, the school sports atmosphere was significantly and negatively correlated with social anxiety (*r* = −0.418, *p* < 0.001). Lastly, exercise self-efficacy demonstrated a strong negative inverse relationship with social anxiety (*r* = −0.482, *p* < 0.001). These foundational bivariate relationships established the statistical prerequisite necessary for advancing to complex meditational modeling.

**Table 2:** Descriptive statistics and correlational matrix for core variables.

Variables	Mean	SD	1	2	3
1. School sports atmosphere	58.62	10.35	1		
2. Exercise self-efficacy	32.47	7.82	0.526^***^	1	
3. Social anxiety	38.54	9.26	−0.418^***^	−0.482^***^	1

^***^p < 0.001.

### 4.3 Testing the mediating effect of exercise self-efficacy

To empirically evaluate Hypothesis 1 and Hypothesis 2, regression-based mediation path analysis was conducted utilizing PROCESS Model 4. Demographic covariates were systematically controlled throughout the equations. The total effect model revealed that the school sports atmosphere was negatively and significantly associated with college students' social anxiety [*B* = −0.372, *SE* = 0.038, *t* = −9.789, *p* < 0.001, 95% CI (−0.447, −0.297)]. This direct negative association provided support for Hypothesis 1.

Subsequent equations evaluated the mediational architecture. The school sports atmosphere was positively associated with the mediator, exercise self-efficacy [*B* = 0.398, *SE* = 0.029, *t* = 13.724, *p* < 0.001, 95% CI (0.341, 0.455)]. When both the independent variable and the proposed mediator were simultaneously regressed onto the dependent variable, exercise self-efficacy was significantly and negatively associated with social anxiety [*B* = −0.425, *SE* = 0.039, *t* = −10.897, *p* < 0.001, 95% CI [−0.502, −0.348)]. Although the direct effect of the school sports atmosphere on social anxiety remained statistically significant in the presence of the mediator [***B*** = −0.203, ***SE*** = 0.041, ***t*** = −4.951, ***p*** < 0.001, 95% CI (−0.284, −0.122)], the absolute magnitude of the coefficient was attenuated.

The application of non-parametric bootstrapping confirmed the statistical significance of the indirect effect. The calculated indirect effect coefficient was −0.169 with a 95% bias-corrected confidence interval of (−0.214, −0.128). Because this interval strictly excluded zero, the presence of a statistically significant mediation effect was established. Specifically, the indirect effect accounted for 45.43% of the total effect mechanism linking the school sports atmosphere to social anxiety, supporting Hypothesis 2.

### 4.4 Testing the moderated mediation model

Building upon the establishment of the foundational mediation framework, PROCESS Model 59 was executed to examine whether gender governed the magnitude of the identified theoretical pathways. In these computations, continuous predictors were mean-centered to eliminate problematic multicollinearity prior to the construction of interaction terms. Gender was coded dichotomously (Male = 0, Female = 1). The complete moderated regression outputs are delineated in Table 3.

**Table 3:** Regression results for the moderated mediation model (PROCESS model 59).

Predictors	Model 1: exercise self-efficacy			Model 2: social anxiety		
	*B*	SE	*t*	*B*	SE	*t*
Constant	32.471	0.321	101.15^***^	38.544	0.279	138.15^***^
School sports atmosphere (SSA)	0.450	0.043	10.46^***^	−0.150	0.042	−3.57^***^
Exercise self-efficacy (ESE)	—	—	—	−0.250	0.055	−4.54^***^
Gender (male = 0, female = 1)	−2.864	0.942	−3.04^**^	3.215	1.204	2.67^**^
SSA × gender	−0.200	0.058	−3.44^***^	0.050	0.082	0.60
ESE × gender	—	—	—	−0.200	0.077	−2.59^**^
Covariates	Controlled			Controlled		
*R^2^*	0.295			0.342		
*F*	31.52^***^			29.47^***^		

^**^p < 0.01, ^***^p < 0.001.

**Figure 2:**
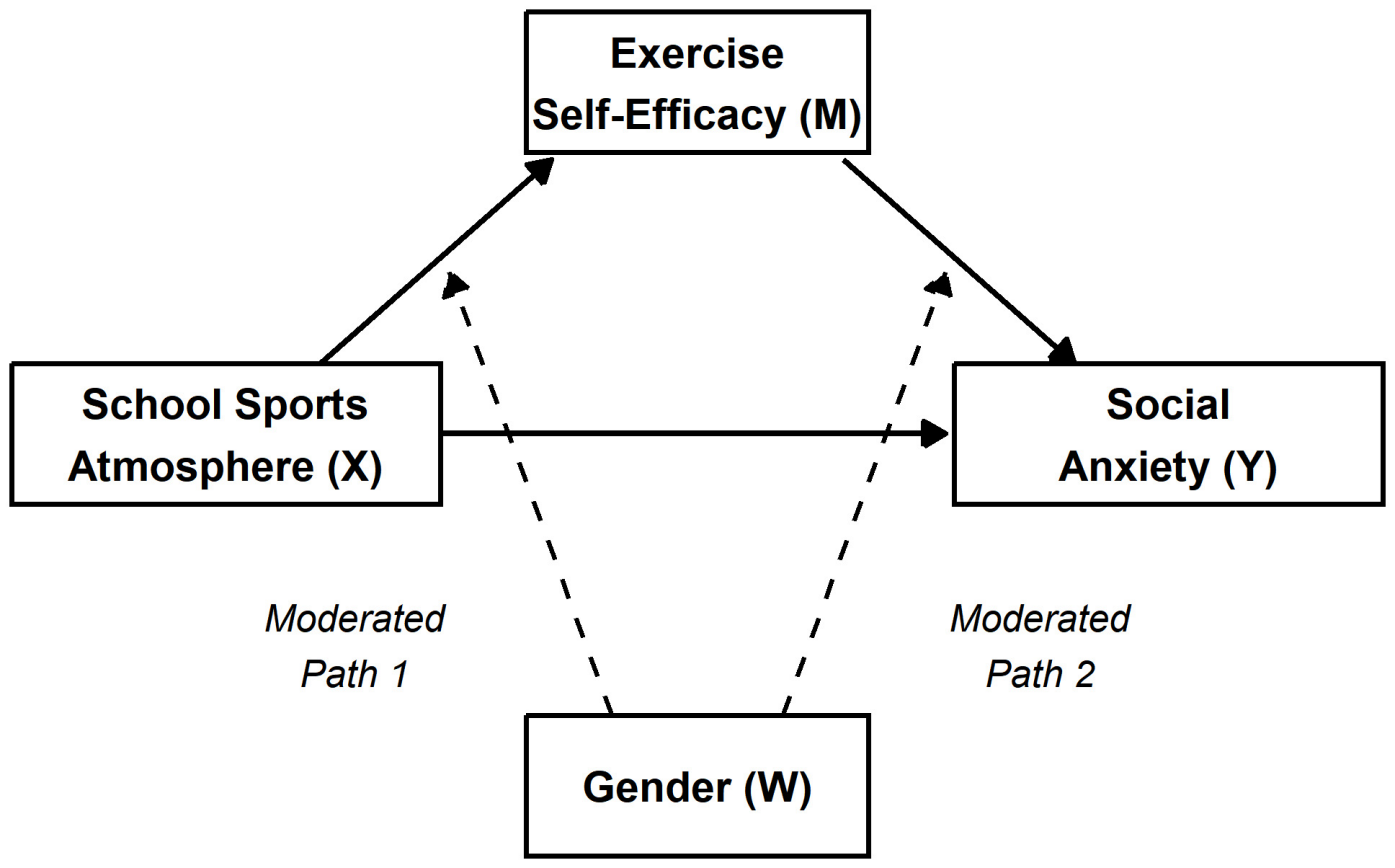
Empirical results model.

The results detailed in Model 1 of Table 3 demonstrate that the interaction term between the school sports atmosphere and gender was significantly and negatively associated with exercise self-efficacy (*B* = −0.200, *t* = −3.44, *p* < 0.001). This confirms that the initial trajectory of the mediational pathway is moderated by demographic gender. Transitioning to Model 2, the analysis revealed that the interaction term bridging exercise self-efficacy and gender was significantly and negatively associated with social anxiety (*B* = −0.200, *t* = −2.59, *p* < 0.01). Conversely, the interaction term between the school sports atmosphere and gender failed to achieve statistical significance in explaining variance in social anxiety (*B* = 0.050, *t* = 0.60, *p* > 0.05), indicating that the residual direct environmental association operates symmetrically across both male and female student populations (as depicted in Figure 2).

**Figure 3:**
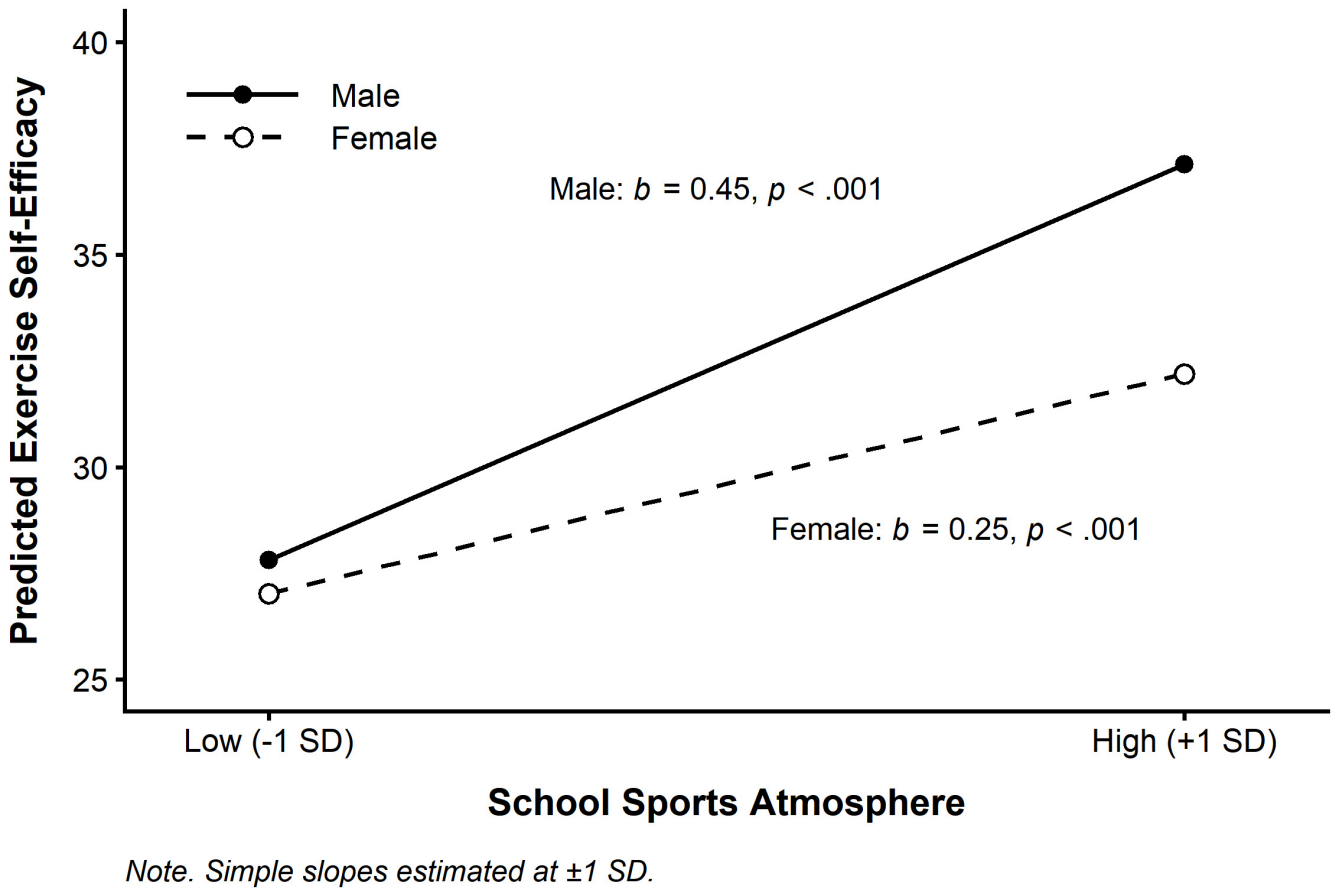
Interaction of school sports atmosphere and gender on exercise self-efficacy. Simple slopes plotted at low (−1 SD) and high (+1 SD) levels of the predictor.

To fully interpret the specific geometry and directionality of these significant interaction effects, simple slope analyses were formulated and plotted at representative conditional values (−1 SD and +1 SD). Regarding the antecedent path (SSA → ESE), the simple slope test revealed that while the school sports atmosphere fostered exercise self-efficacy for all genders, the gradient of this enhancement was steeper for male students (*B*_male_ = 0.450, *p* < 0.001) compared to their female counterparts (*B*female = 0.250, *p* < 0.001). This asymmetric acquisition is visually depicted in Figure 3.

**Figure 4:**
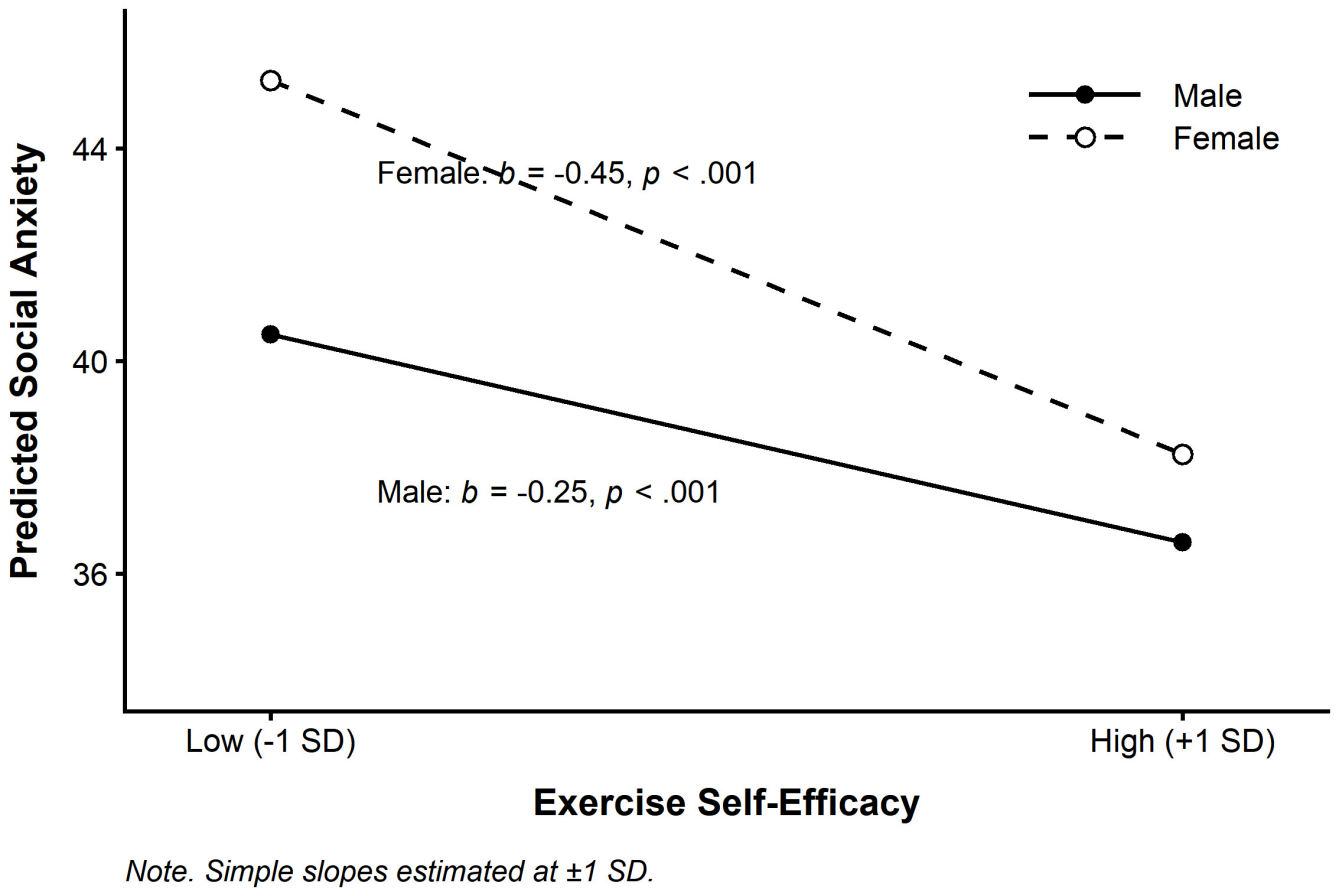
Interaction of exercise self-efficacy and gender on social anxiety. Simple slopes plotted at low (−1 SD) and high (+1 SD) levels of the predictor.

Evaluating the consequent path (ESE → SA), the interaction geometry inverted. For male students, higher exercise self-efficacy was significantly associated with a reduction in social anxiety (*B*_male_ = −0.250, *p* < 0.001). However, for female students, the protective association of exercise self-efficacy against social anxiety was more pronounced (*B*_female_ = −0.450, *p* < 0.001), as illustrated in Figure 4.

Finally, integrating these dual interactions via conditional process analysis illuminated an observed compensatory pattern. The comprehensive conditional indirect effect index for male students was quantified at −0.112 [0.450 × −0.250, 95% CI (−0.165, −0.071)], driven by the initial heightened environmental responsiveness. Concurrently, the index for female students was identically logged at −0.112 [0.250 × −0.450, 95% CI (−0.170, −0.068)], anchored primarily by the amplified second-stage emotional buffering pattern. Consequently, the formal index of moderated mediation assessing the difference between these overall indirect effects was mathematically zero [Index = 0.000, 95% CI (−0.038, 0.041)], indicating the net indirect magnitude does not statistically differ across genders. However, the significant moderation of the constituent paths provides support for Hypothesis 3 in terms of path-specific compensation rather than an overall indirect-effect difference, establishing an observed compensatory tendency where males showed a stronger association between the supportive environment and efficacy acquisition, but females exhibited a stronger negative association between that acquired efficacy and social anxiety.

## 5 Discussion

This empirical investigation leveraged a meticulously controlled moderated mediation paradigm to dissect the complex psychological etiology linking the macro-level school sports atmosphere to the micro-level internal experience of college student social anxiety. The findings enrich the existing intersectional literature surrounding developmental psychology, public health, and higher education administration by corroborating the principal theoretical hypotheses.

### 5.1 The direct protective effect of school sports atmosphere

Consistent with the foundational axioms of Ecological Systems Theory (11), the current data empirically validate that the overarching school sports atmosphere operates as a vital environmental buffer that is negatively associated with college students' social anxiety. This finding corroborates and extends recent epidemiological inquiries highlighting the anxiety-attenuating properties of robust campus recreational ecosystems (14, 40, 41). This aligns with recent systematic evidence indicating that an inclusive physical activity environment is instrumental in disrupting the maladaptive cognitive patterns associated with social evaluation (42, 43). A vibrant, inclusive, and well-resourced sports atmosphere substantially modifies the traditional academic social paradigm. Within a high-stress academic milieu, social interactions are frequently fraught with severe evaluative pressures and intense peer competition, providing fertile ground for the germination of social anxiety.

In contrast, a pervasive sports culture structurally encourages cooperative endeavor, shared physical vulnerability, and ludic engagement. This structured environmental shift may help attenuate the anticipatory threat detection mechanisms inherent in socially anxious cognitive schemas. Furthermore, pervasive environmental cues endorsing physical activity stimulate physiological adaptations across the student body. The facilitation of routine cardiovascular and resistance exercise directly influences the neuroendocrine system, optimizing cortisol regulation and enhancing the proliferation of mood-stabilizing neurotransmitters, thereby establishing a physiological baseline more resistant to anxious arousal (15, 44–46).

### 5.2 The mediating role of exercise self-efficacy

Beyond mere environmental influences, the integration of Bandura's (16) Social Cognitive Theory revealed that the macro-environmental impact is systematically processed through a cognitive mediator: exercise self-efficacy (47). The partial mediation model verified herein illustrates that institutional sporting resources must be translated into personal cognitive agency to achieve meaningful psychological benefit. When educational institutions cultivate a positive sports atmosphere through diverse curricular offerings, state-of-the-art facilities, and encouraging pedagogical methodologies, they are essentially providing a structured repository of the core antecedents of self-efficacy: mastery experiences, vicarious learning, verbal persuasion, and positive physiological appraisal (12, 28, 48).

Once this domain-specific confidence is solidified, a psychological phenomenon known as cross-domain efficacy generalization occurs (20). Recent cross-sectional and longitudinal studies reinforce this, demonstrating that enhanced exercise self-efficacy translates into improved social adaptability and emotional resilience (46). College students who trust their physical resilience and their capacity to conquer exercise-related obstacles may unconsciously apply this empowered cognitive schema to the daunting realm of social interaction. This specific psychodynamic may help alleviate the core clinical feature of social anxiety: the entrenched belief in one's inherent social incompetence and the catastrophic anticipation of peer rejection (37). Consequently, the student may move from a defensive posture of social avoidance to a state of proactive, confident social engagement.

### 5.3 The moderating role of gender and the compensatory pattern

A conceptually novel contribution of this study is the elucidation of the asymmetrical moderating architecture introduced by demographic gender. The statistical outputs demonstrate that while the overarching mediational system is applicable to all genders, its internal mechanics operate distinctively between male and female cohorts, creating a balanced but path-specific variation in the indirect effect.

The first-stage moderation confirmed that males exhibit a heightened sensitivity to the school sports atmosphere, extracting exercise self-efficacy more efficiently than females. This discrepancy is fundamentally rooted in pervasive, culturally ingrained gender socialization frameworks. In many contemporary societies, the masculine identity is intrinsically intertwined with physical prowess and athletic participation (3). Consequently, males approach the campus sports ecosystem with pre-existing athletic schemas and a higher propensity to interpret environmental cues as direct invitations for participation, rapidly consolidating these experiences into self-efficacy. Conversely, females often navigate institutional sports environments burdened by societal stigmas surrounding female athleticism, prevalent body image anxieties, and fears of physical objectification, which dampens the immediate translation of environmental resources into personal efficacy (23).

However, the second-stage moderation revealed a notable compensatory pattern: the association between exercise self-efficacy and the alleviation of social anxiety is more potent for female students. Epidemiological data universally underscore that females represent a significantly higher-risk population for the clinical onset of social anxiety disorders, often exacerbated by intense interpersonal sensitivity and societal pressures regarding physical presentation (24). For a male, possessing high exercise self-efficacy often simply reinforces a culturally normative masculine identity. However, for a female student navigating the psychological complexities of social anxiety, achieving high exercise self-efficacy represents a meaningful psychological benefit. It provides an undeniable counter-narrative to feelings of physical inadequacy and social vulnerability. The acquisition of this physical agency helps overcome restrictive boundaries of social fear, fostering a sense of holistic self-worth that is related to lower interpersonal anxiety.

From a sociological and psychological standpoint, this implies that while the net protective benefit (the overall indirect effect) remains similar across genders, the internal pathway to achieve it is fundamentally different. Males capitalize more readily on external sporting environments to build efficacy, largely due to supportive socialization. Conversely, females face a steeper barrier to gaining this efficacy; yet, once acquired, it serves as a remarkably potent buffer to override their higher baseline vulnerabilities to social anxiety. This highlights a path-specific compensatory pattern rather than a universal mechanism.

### 5.4 Practical implications

The empirical mapping of these psychodynamic pathways yields valuable operational directives for university administrators and clinical counselors. First, addressing social anxiety should not be isolated to the clinical counseling center; it requires a macro-environmental strategy involving the expansion and diversification of the campus sports ecosystem. Institutions should transition toward highly inclusive, recreational, and non-evaluative physical programs that maximize broad participation.

Second, intervention strategies may benefit from being inherently gender-sensitive rather than homogenous. For male populations, the focus should remain on maintaining high environmental accessibility and cultivating positive peer sporting norms to ensure continuous efficacy generation. For female populations, structural environmental exposure may be insufficient; interventions should specifically target the psychological barriers inhibiting the initial formation of exercise self-efficacy (47, 49, 50). This could include the implementation of female-centric or private athletic spaces, body-positive physical education pedagogies, and targeted psychoeducation aimed at dismantling restrictive gender roles in sports, thereby allowing females to access the anxiolytic benefits of physical efficacy.

### 5.5 Limitations and future directions

While the conceptual models and statistical executions provide valuable insights, necessary limitations must be acknowledged. Primarily, the reliance on a cross-sectional dataset fundamentally precludes the establishment of definitive causality. While our directional hypotheses are theoretically grounded, reciprocal causation is possible and cannot be statistically excluded. For instance, debilitating social anxiety may subsequently isolate an individual, lowering their exercise self-efficacy and severing their connection to the campus sports environment (12). Future investigations should prioritize longitudinal cross-lagged panel designs to capture the dynamic temporal sequence of these variables over the academic lifecycle. Furthermore, the reliance on self-report metrics introduces inherent vulnerabilities to social desirability bias, a notable concern when measuring sensitive clinical constructs like anxiety (39). Subsequent research should triangulate self-report data with objective physiological biomarkers of anxiety (e.g., resting heart rate variability) and empirical behavioral tracking of physical activity. Finally, expanding the theoretical model to incorporate parallel mediators such as physical self-esteem and peer attachment security will provide a more granular schematic of this complex socio-psychological architecture.

## 6 Conclusion

In summation, the present research successfully integrated ecological and cognitive theoretical frameworks to illuminate the complex mechanisms through which the school sports atmosphere relates to college students' social anxiety. The findings suggest that a supportive institutional sports environment serves as a protective factor against social anxiety. This macro-environmental association is partially explained through the psychological pathway of exercise self-efficacy. Crucially, this psychodynamic system is significantly shaped by a potential compensatory moderating influence of gender. Males may capitalize more readily on environmental sports resources to build efficacy, whereas females may leverage that acquired efficacy more effectively to manage social anxiety. By recognizing the nuanced, gender-specific mechanisms of cognitive efficacy development, higher education institutions can engineer sophisticated, targeted, and culturally competent psychosocial interventions. Leveraging the physical environment to empower the internal cognitive architecture represents a promising paradigm for promoting the holistic psychological resilience and social flourishing of the contemporary college student population.

## Data Availability

The raw data supporting the conclusions of this article will be made available by the authors, without undue reservation.
